# Dietary ellagic acid inhibiting gastrointestinal pathogens by modulation of microbiome-metabolite-immune axis

**DOI:** 10.1007/s13659-025-00584-x

**Published:** 2026-02-01

**Authors:** Li-Na Mei, Jia-Shan Shen, Yu Duan, Zhuo-Qi Shi, Hui-Zhen Peng, Xiao-Dong Luo

**Affiliations:** 1 Yunnan Characteristic Plant Extraction Laboratory, Key Laboratory of Medicinal Chemistry for Natural Resource, Ministry of Education and Yunnan Province, School of Chemical Science and Technology, Yunnan University, Kunming, 650500 People’s Republic of China; 2 State Key Laboratory of Phytochemistry and Plant Resources in West China, Kunming Institute of Botany, Chinese Academy of Sciences Kunming, Kunming, 650201 People’s Republic of China

**Keywords:** Ellagic acid, Gut microbiota, Short-chain fatty acids, Immune modulation

## Abstract

Antibiotic-induced depletion of the gut microbiota facilitated the colonization of vancomycin-resistant *Enterococci* (VRE) in the gastrointestinal tract, and then increased patients' susceptibility to secondary infections. Ellagic acid, a major constituent of fruits and nuts, showed various bioactivities except for antibacterial. Interestingly, it promoted the recovery of gut microbiota, enhanced microbial diversity and stimulated the proliferation of probiotic gut microbes, and then ameliorated the overgrowth of pathogens in vivo in our experiment. Moreover, ellagic acid activated *Gpr41* and *Gpr43* mainly by promoting the production of short chain fatty acids (SCFAs) such as acetic acid and propionic acid to inhibit the NF-ĸB signaling pathway. Then the dietary supplement with ellagic acid might treat infected gut to avoid antibiotic-associated intestinal diseases, and the finding also provided a novel strategy for exploring antibacterial agent besides screening in vitro.

**Graphical Abstract:**

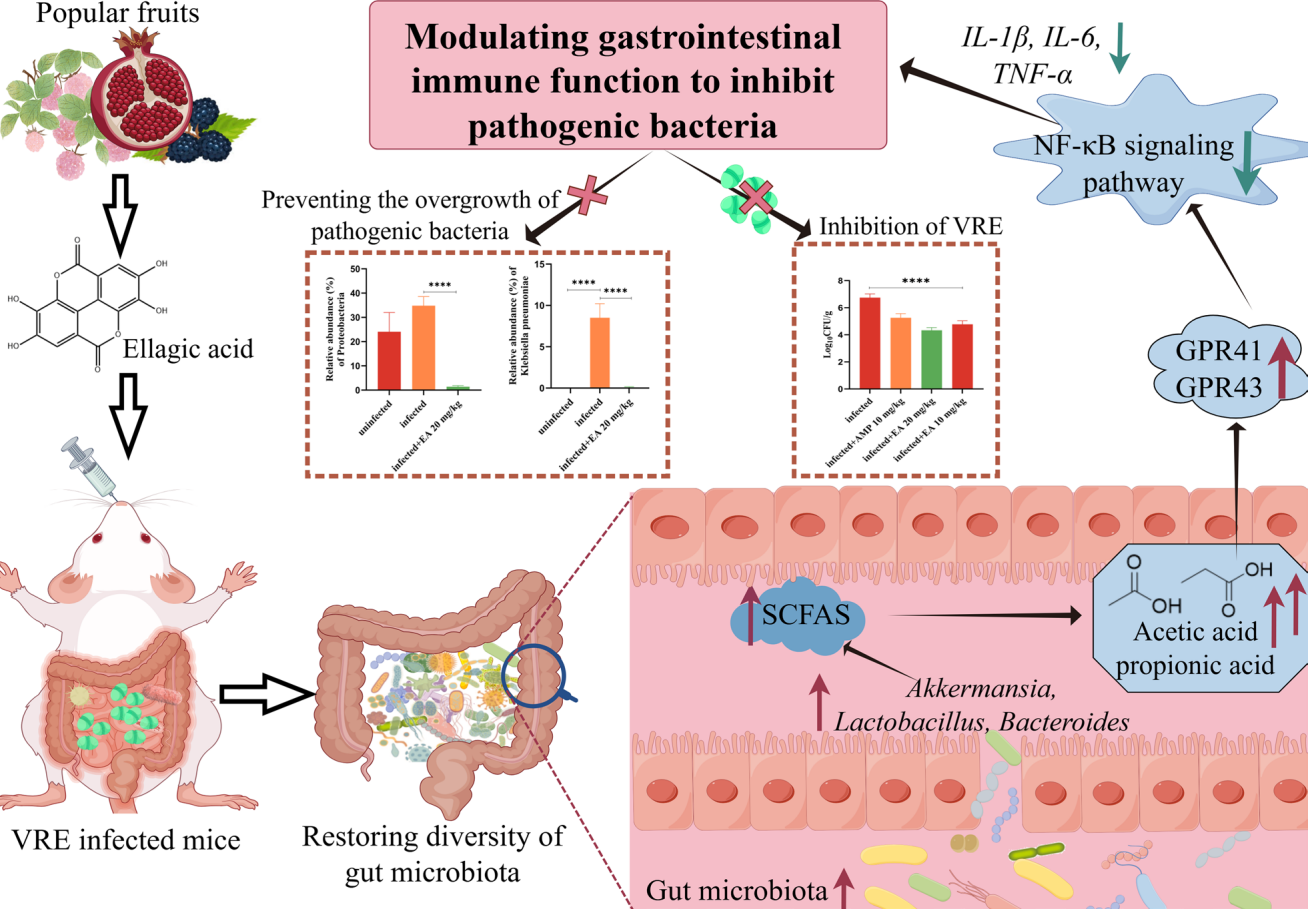

**Supplementary Information:**

The online version contains supplementary material available at 10.1007/s13659-025-00584-x.

## Introduction

Vancomycin-resistant *Enterococcus* (VRE), a major nosocomial pathogen, can persistently colonize the gastrointestinal tract, potentially causing severe infections such as bacteremia, sepsis, and urinary tract infections [1–3]. Currently, antibiotics such as amoxicillin, ampicillin (AMP), linezolid, daptomycin, nitrofurantoin, and fosfomycin are recommended as first-line treatments for VRE infection [4]. However, the overuse of antibiotics always result in dysbiosis and depletion of the intestinal microbiome, inhibiting sensitive and beneficial bacterial populations [5]. This imbalance might provide a and survival advantage to drug-resistant and opportunistic pathogens, further complicating the microbial ecosystem and leading to adverse health outcomes [6–8]. Prolonged use of vancomycin (VAN) may result in a compensatory increase of Gram-negative Proteobacteria and VAN-resistant bacilli, along with decreased peripheral insulin sensitivity [9, 10]. Notably, VAN could trigger *Klebsiella pneumoniae* outgrowth in a subset of outgrowth-susceptible animals [11]. Furthermore, the human enteropathogen *Escherichia albertii* might be more abundant after azithromycin treatment, which contribute to the onset of diarrhea in patients [12]. Similarly, prolonged post-antibiotic dysbiosis in mice rendered them susceptible to infection by the intestinal pathogenic bacteria, such as VRE [13]. Emerging solutions to antibiotic-driven ecological disruptions, such as enteric dysbiosis, target immune system reconfiguration to bolster innate infection resistance, offering a viable pathway to decrease antibiotic dependence [14]. Fecal microbiota transplantation mediates disease treatment by reestablishing gut microbial balance and immune regulation via transplanted microbiota and their functional metabolites [15]. However, emerging evidence suggests that this procedure may induce profound modifications in enteric ecosystem dynamics, with adverse consequences for metabolic function, neurological-behavioral outputs, and energy balance regulation [16]. This underscores the necessity of developing safer, affordable alternatives for microbiome rehabilitation that minimize off-target physiological consequences.

Ellagic acid, a dietary polyphenolic compound, is abundant in strawberries, red and black raspberries, pecans, pomegranates, and grapes [17–20], which was reported with concentrations of up to 570 mg/100 mL in pomegranate juice, 47–90 mg/g (dry weight) in red and black raspberries, and 21–86 mg/g (dry weight) in pecans [21, 22]. It exhibited a broad spectrum of pharmacological activities, including antioxidative, anti-inflammatory, anticancer, hepatoprotection, neuroprotection, dermatoprotection, cardiovascular protection, and immune modulation [23–27]. Surprisingly, in this paper, ellagic acid did not show antibacterial properties in vitro*,* but it restored the composition of the infected gut microbiota and enhanced the immune defense of the host against pathogens in vivo.

## Materials and methods

**Experimental Materials and Equipment.** Ellagic acid was sourced from Macklin Biochemical Technology Co., Ltd. (Shanghai, China). Isopropyl ether, phosphoric acid, and additional reagents were obtained from China National Pharmaceutical Group Corporation (China). For SCFAs analysis, a gas chromatography system (7890B GC System, Agilent Technologies, USA) coupled with a mass spectrometer (5977B GC/MSD, Agilent Technologies, USA) was employed. For PCR analysis, the following instruments were utilized: a spectrophotometer (BioPhotometer® D30, Eppendorf, Germany), a gradient PCR instrument (Mastercycler® nexus GX2, Eppendorf, Germany), and a LightCycler® 96 Instrument (Roche, Switzerland).

**Animal Ethics in Research and Practice.** The experimental subjects were female Kunming strain mice (18—20 g), which were specific pathogen-free (SPF). The mice were procured from the Experimental Animal Center of Yunnan University and acclimatized for one week prior to the experiment under controlled conditions (25 °C, 40%-60% relative humidity). The study protocol was approved by the Laboratory Animal Welfare and Ethics Committee of Yunnan University (certificate number: YNU20241078; approval date: October 15, 2024).

**Bacterial Strains and Their Corresponding Cultivation Conditions**. The VRE, *Enterococcus faecalis*, *Staphylococcus aureus*, *Escherichia coli*, and *P. mirabilis* strains were cultured on tryptic soy agar (TSA). A single colony was selected from the TSA plate and inoculated into tryptic soy broth (TSB). The inoculated TSB was incubated at 37 ℃ with a shaking speed of 180 revolutions per minute (rpm) for 12 h to obtain a bacterial suspension in the logarithmic growth phase.

**Murine model**. To evaluate the impact of ellagic acid on antibiotic-induced dysbiosis, the gut microbiota was initially depleted using antibiotics, and a VRE infection model was established to simulate a pathogenic bacterial infection environment [28]. In the experiment, all mice were administered drinking water containing vancomycin (VAN, 500 mg/mL) and AMP (500 mg/mL) for one week to induce intestinal microbiota disruption. To maintain antibiotic potency, the drinking water was replaced every two days. On day 0 of the experiment, the mice were inoculated with VRE (10^9^ CFU/mL) after a 4-h fasting period to establish an infection model. Subsequently, the mice were randomly divided into five groups (n = 7 per group): uninfected control, infected control, AMP treatment, ellagic acid high-dose, and ellagic acid low-dose. The detailed experimental methods were provided in Supporting Information 2.1.

**Analysis of Gut Microbiota**. Genomic DNA was extracted from fecal samples of the uninfected group, infected group, and 20 mg/kg ellagic acid group using either CTAB or SDS methods. After processing, sequencing was performed on the NovaSeq6000 platform, followed by comprehensive data analysis [29–31]. For detailed experimental procedures, please refer to Supporting Information 2.2.

**Analysis of SCFAs constituents**. Serum samples were collected from the uninfected, infected, and 20 mg/kg ellagic acid groups. A measured volume of each sample was mixed with 50 µL of 20% phosphoric acid, and then an isopropyl ether solution containing 500 µM internal standard was added. The mixture was vigorously vortexed and centrifuged at 14,000 rpm for 20 min at 4 ℃ [32]. The instrumental parameters for gas chromatography-mass spectrometry (GC–MS) were comprehensively documented in the Supporting Information 2.3.

**Quantitative real-time reverse transcription-PCR (RT-qPCR)**. *Gpr41* and *Gpr43* expression was evaluated by RT-qPCR, and the gene-specific primer information of *Gpr41*, *Gpr43*, and *β-actin* (the control gene) was detailed in Table S3. Experimental methods were provided in Supporting Information 2.4.

**Biochemical indices of the mouse cecum**. The expression levels of *NF⁃κB p65, IL-6, IL-1β,* and *TNF-α* in the cecum of mice were quantified following the methods described in the previous section, and the primer sequences for these cytokines are listed in Table S3.

**Antibacterial bioactivity of ellagic acid in vitro.** The inhibitory bioactivity of ellagic acid against VRE, *Enterococcus faecalis*, *Staphylococcus aureus*, *Escherichia coli*, and *Proteus mirabilis* was evaluated in vitro using broth microdilution method. Bacterial cultures were incubated at 37 °C with shaking at 180 rpm overnight and subsequently diluted to a concentration of 2 × 10^5^ CFU/mL. Aliquots of these suspensions were transferred into 96-well plates containing varying concentrations of ellagic acid (512—1 µg/mL) with a final volume of 100 µL per well. AMP, VAN, polymyxin B, and kanamycin were used as positive controls at equivalent concentrations, while untreated bacterial suspensions served as negative controls. All samples were incubated at 37 °C for 24 h. MIC was defined as the lowest concentration of the drug that completely inhibited visible bacterial growth.

**Statistical analysis:** All data in this study were independently replicated at least three times and were expressed as the mean ± standard deviation. Statistical analysis was conducted using GraphPad Prism 10.1.2 software, with comparisons between groups performed using T-test and one-way analysis of variance. *P* > 0.05 was considered as non-significant (ns), while * *P* < 0.05, ** *P* < 0.01, *** *P* < 0.001, and **** *P* < 0.0001 were considered as statistically significant.

## Results

**Fig. 1:**
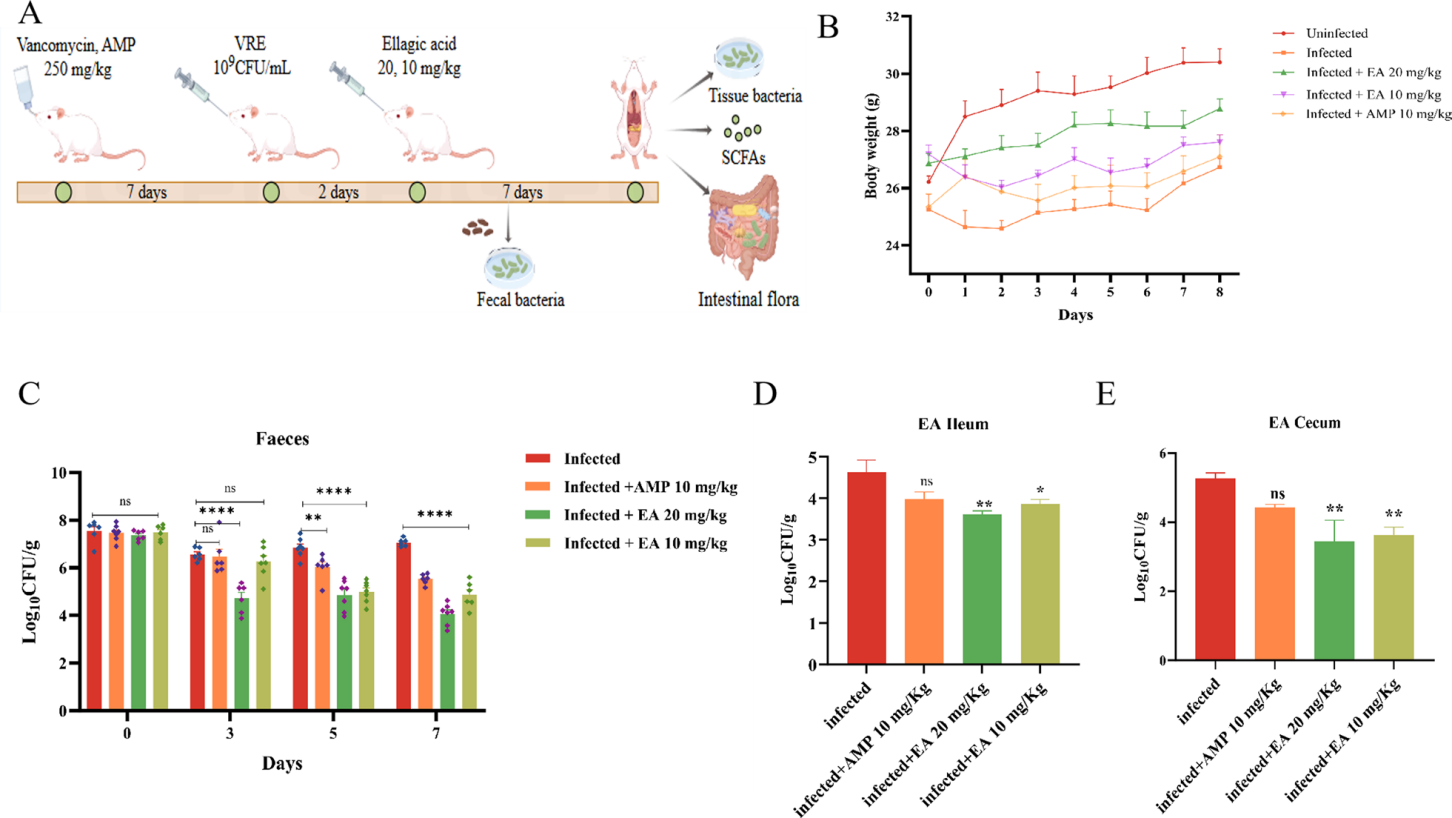
Ellagic acid inhibiting VRE colonization in vivo. **A** Flowchart of the mouse infection model. **B** Changes in body weight of mice during the experiment. **C** VRE load in fecal samples. **D** VRE load in the ileum of mice. **E** VRE load in the cecum of mice. "ns" indicates no statistically significant difference. *P < 0.05 versus Infected group; **P < 0.01 vs. Infected group; ****P < 0.0001 vs. Infected group. EA: Ellagic acid

**The capability of ellagic acid decolonizing VRE in vivo**. The experimental procedure for the mouse model was illustrated in Fig. 1A. Subsequent to VRE infection, the mice initially exhibited weight loss, which was progressively reversed with ellagic acid treatment, reverting to normal growth patterns by the fourth day. However, the infected group did not show significant weight gain until the sixth day (Fig. 1B). Analysis of fecal bacterial load showed a substantial, dose-dependent reduction in gastrointestinal VRE colonization after ellagic acid administration (Fig. 1C). Following successful infection, the VRE fecal loads on day 1 were as follows (Log₁₀ CFU/mL): infected group, 7.54; AMP group (10 mg/kg), 7.47; ellagic acid (20 mg/kg), 7.36; ellagic acid (10 mg/kg), 7.49. By day 3, bacterial counts decreased by 0.99 Log₁₀ CFU/mL in the infected group, compared to a reduction of 2.64 Log₁₀ CFU/mL in the 20 mg/kg ellagic acid group. In contrast, the reductions in the 10 mg/kg AMP group (1.00 Log₁₀ CFU/mL) and the 10 mg/kg ellagic acid group (1.23 Log₁₀ CFU/mL) were not significantly different from that in the infected group. From day 4 onward, the bacterial count in the infected group remained stable, showing no significant difference from the baseline (day 1). Notably, by the end of the treatment period, the 20 mg/kg and 10 mg/kg ellagic acid groups achieved reductions of 3.29 and 2.62 Log₁₀ CFU/mL, respectively. Furthermore, the 10 mg/kg ellagic acid group showed superior efficacy to the 10 mg/kg AMP group, with an additional reduction of 1.94 Log₁₀ CFU/mL. The ileal bacterial load in mice (Fig. 1D) indicated lower bacterial loads in the ellagic acid treatment groups. The bacterial load in the infected group was 4.63 Log_10_ CFU/mL, while the ellagic acid 20 mg/kg and 10 mg/kg groups showed reduction of 1.0 Log_10_ CFU/mL and 0.75 Log_10_ CFU/mL, respectively, even better than AMP group. Similarly, in cecal tissue (Fig. 1E), the ellagic acid treatment groups were more effective than the AMP group, with a significant reduction in VRE colonization of the intestinal tract of mice.

**Fig. 2:**
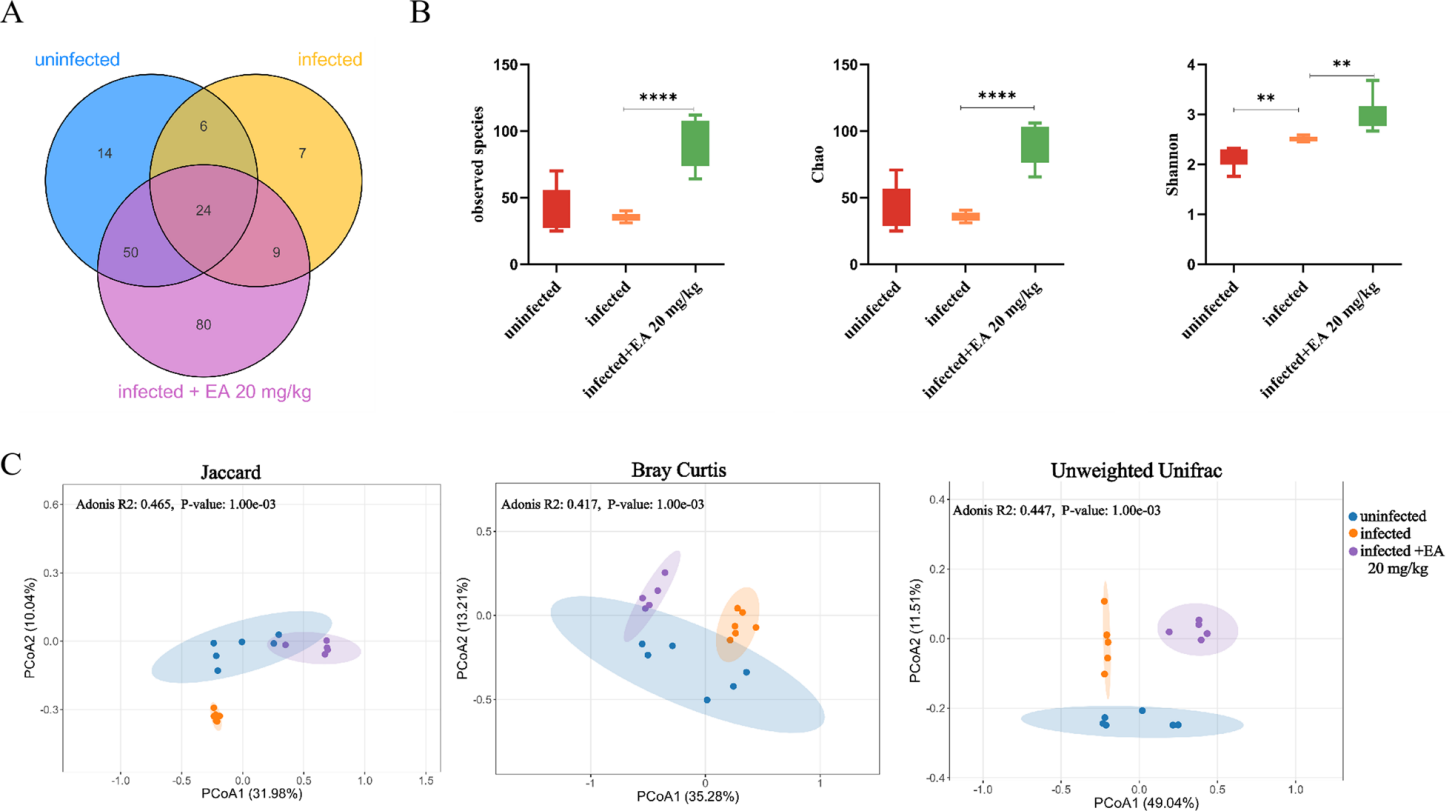
Ellagic acid (20 mg/kg) altered the diversity of the gut microbiota significantly. **A** Venn diagram illustrating the distribution of ASVs in the gut microbiota of mice across different groups. **B** α-diversity metrics, including the Chao1 index, observed species, and Shannon index. **C** PCoA plots at the genus level, based on Jaccard distance, Bray–Curtis distance, and unweighted UniFrac distance algorithms. **P < 0.01 vs. Infected group; ****P < 0.0001 versus Infected group. EA: Ellagic acid

**The impact of ellagic acid on the intestinal microbiota in mice**. High-throughput sequencing of the 16 S rRNA gene was conducted on fecal bacterial DNA extracted from uninfected, infected, and 20 mg/kg ellagic acid-treated to investigate their influence on the gut microbiome. The infected group exhibited 46 unique amplicon sequence variants (ASVs), a significant reduction comparison to the uninfected group (94 ASVs). Interestingly, the 20 mg/kg ellagic acid group showed a substantial increase, with 163 ASVs. (Fig. 2A). Compared to the uninfected group, the infected group showed lower Chao1 and observed species indices; however, treatment with 20 mg/kg ellagic acid reversed this trend, resulting in significantly higher α-diversity (Fig. 2B). β-diversity analysis was then performed, and principal coordinate analysis (PCoA) plots were generated using Jaccard distance, Bray–Curtis distance, and unweighted UniFrac distance algorithms. The obvious separation of ASVs among the three groups indicated distinct community structures, highlighting differences in their microbial composition (Fig. 2C). The results suggested that ellagic acid (20 mg/kg) not only restored the reduced diversity of the gut microbiota but also modulated its structural composition.

**Fig. 3:**
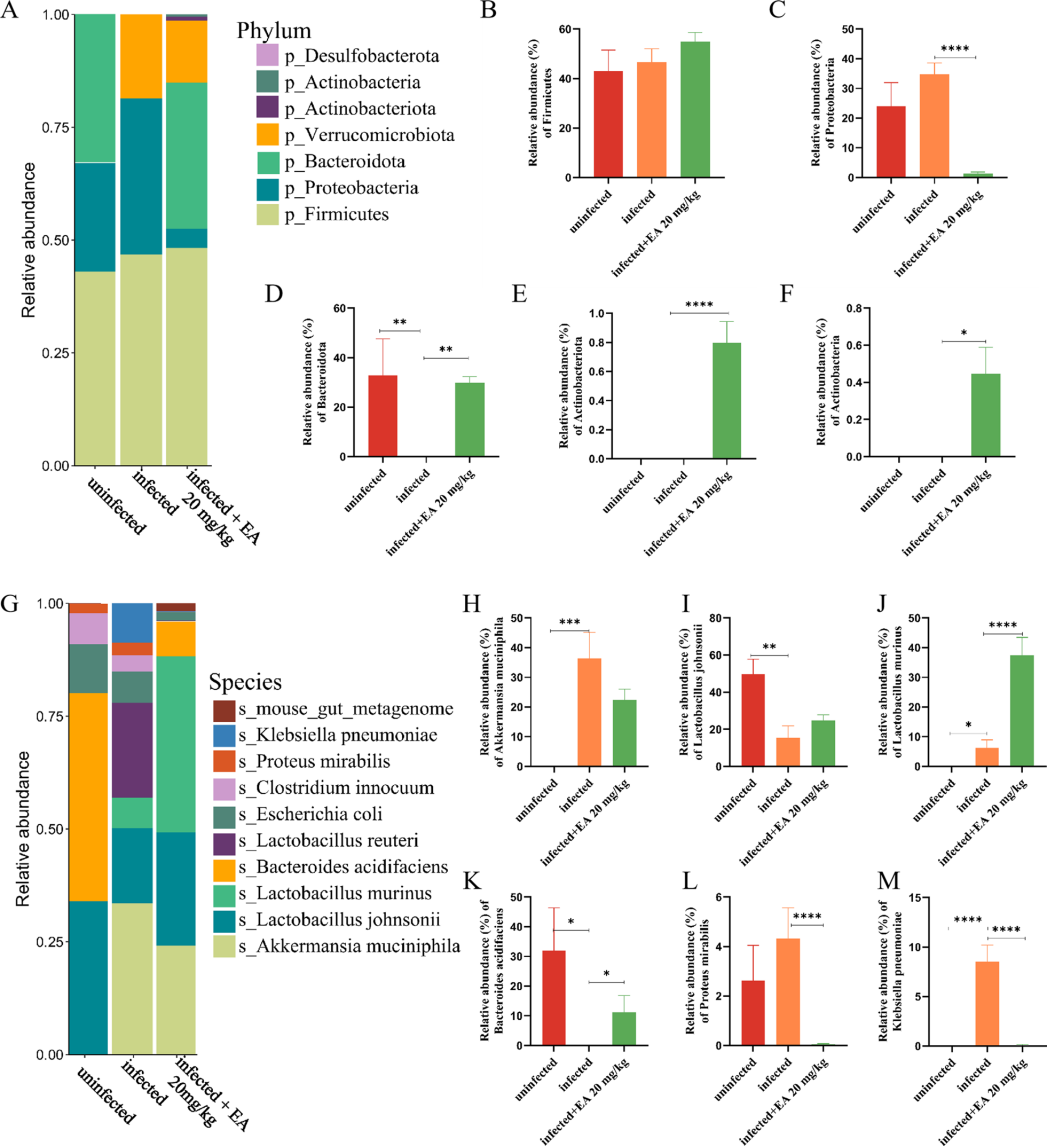
Ellagic acid (20 mg/kg) treatment altered the composition of the gut microbiota. **A** Bar plots showing the relative abundance at the phylum level in the uninfected, infected, and ellagic acid groups. The relative abundances of *Firmicutes* (**B**)*, Proteobacteria* (**C**)*, Bacteroidota* (**D**)*, Actinobacteriota* (**E**), and *Actinobacteria* (**F**) in the uninfected, infected, and ellagic acid groups (**G**) Bar plots showing the relative abundance at the species level in the uninfected, infected, and ellagic acid groups. The relative abundances of *Akkermansia muciniphila* (**H**), *Lactobacillus johnsonii* (**I**), *Lactobacillus murinus* (**J**), *Bacteroides acidifaciens* (**K**), *Proteus mirabilis* (**L**), and *Klebsiella pneumoniae* (**M**) in the uninfected, infected, and ellagic acid groups. “ns” indicated no statistically significant difference. *P < 0.05 versus Infected group; **P < 0.01 versus Infected group; ***P < 0.001 versus Infected group; ****P < 0.0001 versus Infected group. EA: Ellagic acid

The uninfected group exhibited the highest abundances of *Firmicutes*, *Proteobacteria*, and *Bacteroidota* at the phylum level, accounting for 43%, 24% and 33% of the gut microbiota, respectively. In contrast, the infected group showed an increased abundance of *Proteobacteria*, while *Bacteroidota* was undetectable (Fig. 3A). Following drug intervention, ellagic acid (20 mg/kg) increased the abundance of *Firmicutes* (Fig. 3B), reduced the abundance of *Proteobacteria* (Fig. 3C) and reversed the effect by increasing the abundance of *Bacteroidota* (Fig. 3D). Additionally, the ellagic acid enhanced microbial diversity (Fig. 3E, F), with notable increases in the abundances of *Actinobacteriota* and *Actinobacteria* comparison to the uninfected and infected groups. At the species level (Fig. 3G), the ellagic acid group exhibited higher abundances of beneficial bacteria such as *Akkermansia muciniphila* (Fig. 3H), *Lactobacillus johnsonii* (Fig. 3I), *Lactobacillus murinus* (Fig. 3J), and *Bacteroides acidifaciens* (Fig. 3K), accounting for 11%, 37%, 24%, and 22% of the gut microbiota, respectively. Furthermore, ellagic acid reversed the dysregulated trends observed in *L. johnsonii* and *B. acidifaciens* abundances. Notably, ellagic acid treatment also reduced or eliminated pathogens such as *Clostridium innocuum*, *Proteus mirabilis* (Fig. 3L), and *Klebsiella pneumoniae* (Fig. 3M), which were elevated in the infected group. These findings suggest that ellagic acid helps restore gut microbial homeostasis.

**Fig. 4:**
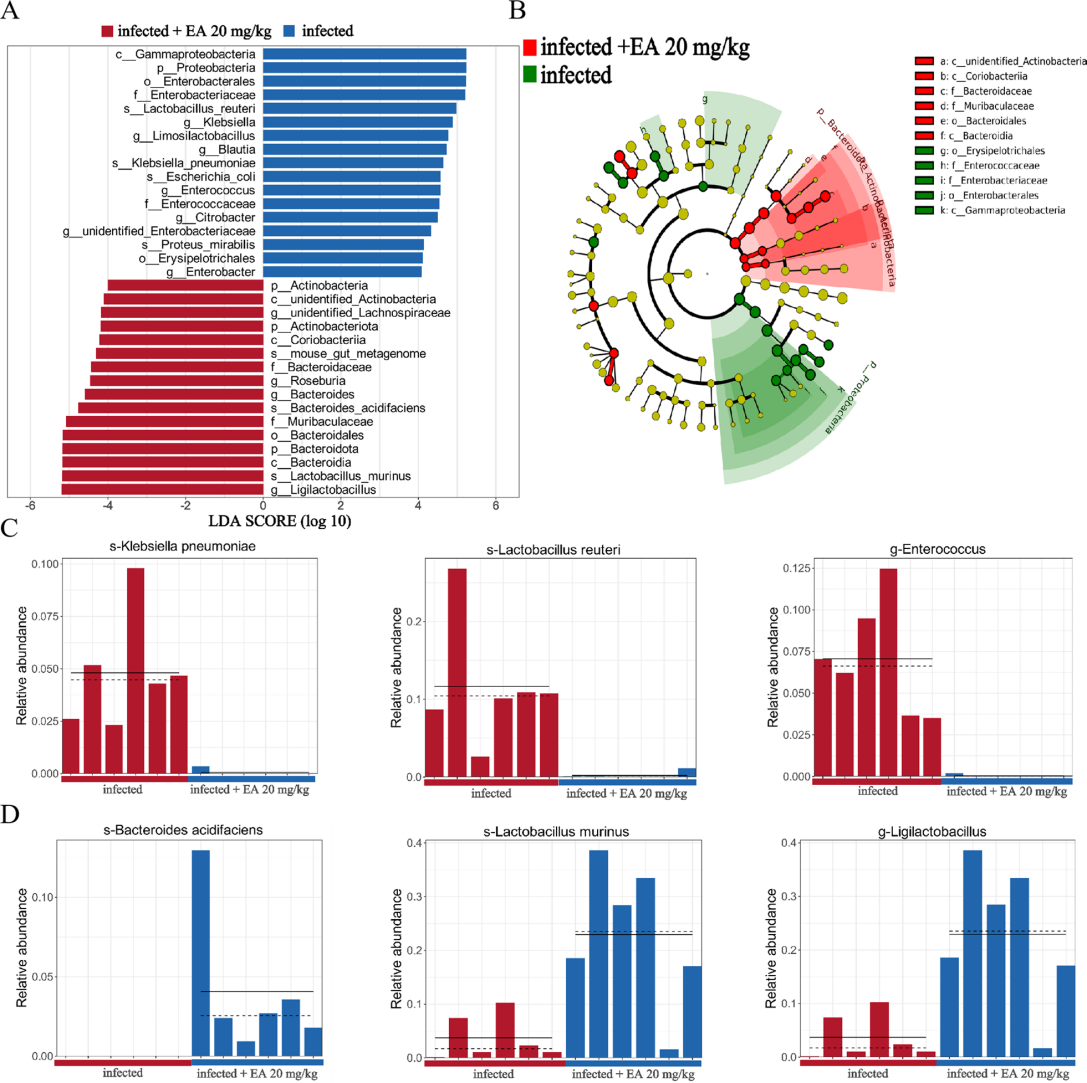
LEfSe analysis between the infected and ellagic acid (20 mg/kg) groups. **A** LDA score plot showing differentially abundant taxonomic features between the infected and ellagic acid groups. **B** Cladogram based on ASVs illustrating the phylogenetic distribution of microbial taxa in the infected and ellagic acid groups. **C** Dominant bacterial taxa in the infected group. **D** Dominant bacterial taxa in the ellagic acid treatment group. EA: Ellagic acid

Linear discriminant analysis Effect Size (LEfSe) were conducted (LDA > 4, *P* < 0.05) to further elucidate the predominant bacterial biomarkers. 18 taxonomic groups of dominant bacterial markers were identified in the infected group, whereas 16 such groups were presented in the ellagic acid-treated group (20 mg/kg) (Fig. 4A, B). *K. pneumoniae* (spanning from *Proteobacteria* to *Klebsiella*), *L. reuteri*, and *Enterococcus* were identified as the predominant bacteria in the infected group (Fig. 4C), which were likely associated with the exacerbation of inflammation and the establishment of VRE colonization in the host. Conversely, *B. acidifaciens*, *L. murinus*, and *Ligilactobacillus* were identified as the dominant bacteria after ellagic acid treatment (Fig. 4D), indicating its property against VRE and other pathogenic bacteria in vivo.

**Fig. 5:**
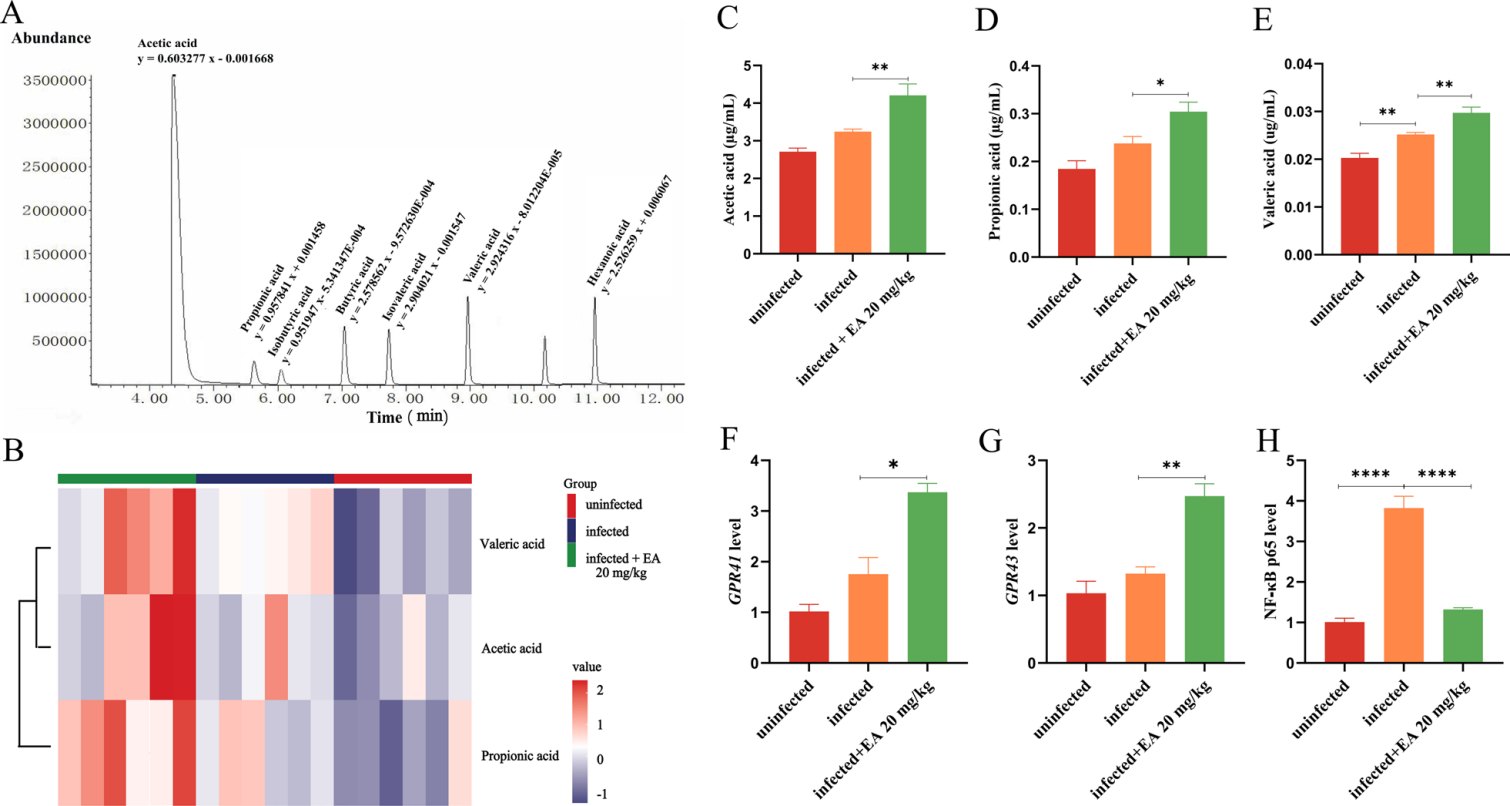
Ellagic acid (20 mg/kg) treatment increasing the concentration of microbial metabolite SCFAs. **A** XIC (extracted ion chromatogram) of standard mixture and linear equations. **B** Heatmap of differential SCFAs after ellagic acid treatment. **C** Concentration of acetate. **D** Concentration of propionate. **E** Concentration of valerate. **F** Expression level of Gpr41. **G** Expression level of* Gpr43*. **H** Expression level of *NF-ĸB p65*. EA: Ellagic acid

**Impact of ellagic acid on short-chain fatty acids**. SCFAs are well documented products of microbial metabolism and affect the host directly, separate metabolomic analyses were performed to quantify absolute levels of SCFAs in serum samples from mice. The quantitative parameters along with their corresponding linear ranges for the target metabolites were established systematically (Fig. 5A, Table S1). As shown in Fig. 5B, the principal differential metabolites after ellagic acid treatment were acetic acid, propionic acid, and valeric acid. Compared to the infected group, their levels increased by 29.7%, 28.0%, and 17.9%, respectively (Fig. 5C–E).

**The impact of ellagic acid on Gpr41 and Gpr43**. The increased levels of SCFAs can activate GPCRs (e.g., GPR41 and GPR43), which regulate immune responses and ameliorate disease pathogenesis. As a result, ellagic acid significantly enhanced the expression levels of *Gpr41* and *Gpr43*, which were 3.37-fold and 2.47-fold higher than those in the uninfected group, respectively. Furthermore, it can recover the expression of *NF-ĸB p65* to normal (Fig. 5F–H).

**Fig. 6:**
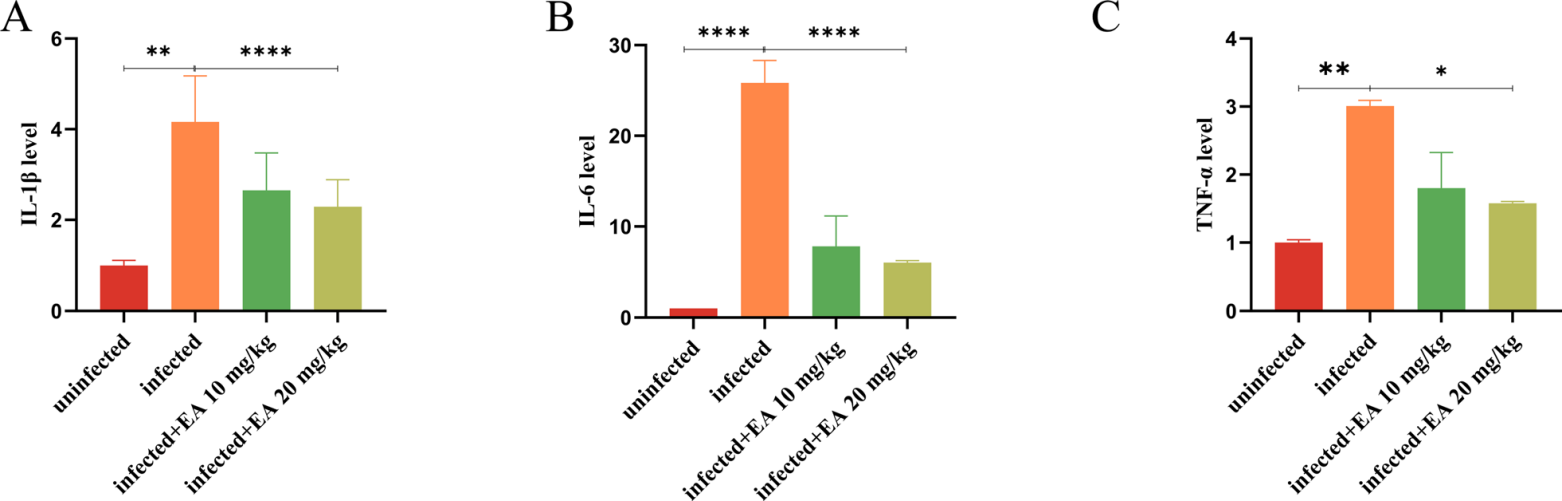
Effects of ellagic acid on pro-inflammatory factors in mice. **A** The expression of *IL-1β*. **B** The expression of *IL-6*. **C** The expression of *TNF-α*. EA: Ellagic acid

**Impact of ellagic acid on the expression of proinflammatory factors in mice**. The inflammatory micro-environment could be exacerbated by bacterial infection, and the expression levels of *IL-1β*, *IL-6*, and *TNF-α* in the infected group were increased significantly (Fig. 6A–C). As expected, ellagic acid reduced the expression of these pro-inflammatory cytokines to modulate NF-κB signaling, suppress the inflammatory mediators and mitigate the inflammatory response.

**Evaluation antibacterial bioactivity of ellagic acid in vitro.** Antibacterial assay was carried out to determine antibacterial property of ellagic acid in vitro, and the results showed its non-activity against all the 5 tested strains. The minimum inhibitory concentration (MIC) values were detailed in Table S2.

## Discussion

Microbiome dysbiosis caused by antibiotic treatment [33] or diarrheal illness [34] might trigger opportunistic infection following the perturbation, and window of susceptibility was extended by dietary supplement of ellagic acid. In our investigation, it modulated the microbiota-metabolite-immune axis, and suppressed the invasion of foreign pathogens and the overgrowth of opportunistic pathogens within the host.

A reduction in the diversity of intestinal microbiota species could increase susceptibility to colonization by harmful pathogens, and stimulate the development of antibiotic resistance in bacteria [35]. Antibiotic interference reduced the diversity of the gut microbiota in mice significantly, which created favorable conditions for VRE colonization and overgrowth of other pathogenic bacteria. Now, ellagic acid could restore gut microbiota diversity significantly with a notable increase in the abundance of beneficial bacteria. The genus *Lactobacillus*, recognized as a group of probiotics, plays a beneficial role in various disease processes, in which *L. johnsonii* and *L. murinus* can inhibit the growth of pathogenic bacteria through multiple mechanisms [36, 37]. Additionally, some Bacteroides strains may produce bacteriocin-like substances to inhibit or kill pathogenic bacteria directly [38, 39]. On the other hand, *C. innocuum*, *P. mirabilis,* and *K. pneumoniae* are opportunistic pathogens that could induce severe, life-threatening infections frequently in their hosts, including pneumonia, bacteremia, and complex urinary tract infections [40–42]. Interestingly, ellagic acid without antibacterial bioactivity in vitro*,* suppressed the overgrowth of these pathogenic bacteria by an in-depth analysis of the gut microbiota. Ellagic acid can facilitate the proliferation of beneficial bacteria, consequently mitigating liver damage associated with non-alcoholic fatty liver disease [43]. In our investigation, it promoted the growth of beneficial bacteria, such as* L. johnsonii* and *L. murinus,* to prevent the overdevelopment of pathogenic bacteria.

SCFAs are key metabolites produced by gut microbiota, such as *Bifidobacterium* and *Lactobacillus* could generate SCFAs through the anaerobic fermentation of substrates like dietary fibers [44]. In this paper, ellagic acid increased the abundance of beneficial bacteria such as *Akkermansia*, *Lactobacillus*, and *Bacteroides*, and then promoted the production of acetic acid, propionic acid and valeric acid.

SCFAs interacts with the immune system through multiple pathways [45]. For example, propionate could inhibit Th17 cell differentiation to mitigate central nervous system inflammation and suppresses histone deacetylase bioactivity, and then enhance immune regulation [26]. Acetate or propionate could activate important SCFA receptors (GPR41 or GPR43) to modulate immune cell function [46]. Now, *Gpr41* and *Gpr43* were activated, and then suppressed the NF-κB signaling pathway and led to a reduction in the expression of pro-inflammatory factors. NF-κB is a critical transcription factor that controls the expression of numerous genes involved in innate and adaptive immune responses [47], so ellagic acid might play a central role in mediating microbiome reconstitution and subsequent immune function modulation.

In summary, ellagic acid, by promoting the proliferation of beneficial commensal bacteria, may indirectly reduce the survival space and nutritional resources available for VRE. Additionally, EA could help create an intestinal microenvironment that is unfavorable for VRE colonization and persistence by enhancing intestinal barrier function and modulating host innate or adaptive immune responses, thereby aiding in the clearance of the pathogen.

## Supplementary Information

Additional file1 (DOCX 23 kb)

## Data Availability

Gut microbiome data and short-chain fatty acids data were deposited at Mendeley Data (Mendeley Data) with reserved DOI (10.17632/47zsbmxx2v.1) and accessed URL (ellagic acid inhibiting gastrointestinal pathogens—Mendeley Data). Source data are provided with this paper and supporting material.
