# Supplementary material for: Dietary ellagic acid inhibiting gastrointestinal pathogens by modulation of microbiome-metabolite-immune axis
Source: Nat Prod Bioprospect. 2026 Feb 1;16(1):21. doi: 10.1007/s13659-025-00584-x (PMC12860781; doi:10.1007/s13659-025-00584-x)
Supplement: Supplementary file 1 — Additional file1 (DOCX 23 kb) [file 13659_2025_584_MOESM1_ESM.docx]

**Supporting Information**

**Dietary ellagic acid inhibiting pathogens through the microbiota-metabolite-immune axis**

LI-NA MEI ^a^, JIA-SHAN SHEN ^a^, YU DUAN ^a^, ZHUO-QI SHI ^a^, HUI-ZHEN PENG ^a^, XIAO-DONG LUO ^a, b, *^

*^a^* *Yunnan Characteristic Plant Extraction Laboratory, Key Laboratory of Medicinal Chemistry for Natural Resource, Ministry of Education and Yunnan Province, School of Chemical Science and Technology, Yunnan University, Kunming, 650500, P. R. China.*

*^b^ State Key Laboratory of Phytochemistry and Plant Resources in West China, Kunming Institute of Botany, Chinese Academy of Sciences Kunming, 650201, P.R. China.*

______________________________________

*** Corresponding Author:**

***Prof. Dr. Xiao-Dong Luo***

Tel: +86-0871-65223177

Email: [xdluo@mail.kib.ac.cn](mailto:xdluo@mail.kib.ac.cn)

**Results**

*1.1. Impact of* ellagic acid *on short chain fatty acids (SCFAs)*

**Table S1 Demonstration of Quantitative Parameters for SCFAs**

| Metabolite Name | Retention Time  (min) | Linear | R^2^ |
| --- | --- | --- | --- |
| Acetic acid | 4.2781 | Y = 0.603277 X - 0.001668 | 0.99 |
| Propionic acid | 5.3333 | Y = 0.957841 X + 0.001458 | 0.99 |
| Butyric acid | 6.6785 | Y = 2.578562 X - 9.572630E-004 | 0.99 |
| Isobutyric acid | 5.7203 | Y = 0.951947 X - 5.341347E-004 | 0.99 |
| Valeric acid | 8.6529 | Y = 2.924316 X - 8.012204E-005 | 0.99 |
| Isovaleric acid | 7.3718 | Y = 2.904021 X - 0.001547 | 0.99 |
| Hexanoic acid | 10.8309 | Y = 2.526259 X + 0.006067 | 0.99 |

*1.2. Ellagic acid in vitro antibacterial bioactivity*

**Table S2. *In vitro* antibacterial bioactivity of** ellagic acid

| Bacterial strains | ellagic acid MIC (µg/mL) | Positive control MIC (µg/mL) |
| --- | --- | --- |
| VRE (ATCC51299) | > 512 | AMP = 4 |
| *Enterococcus faecalis* (SC009) | > 512 | AMP = 2 |
| *Staphylococcus aureus* (ATCC25923) | > 512 | VAN = 2 |
| *Escherichia coli* (ATCC43888) | > 512 | Polymyxin B = 1 |
| *Proteus mirabilis* (CMCC49005) | > 512 | Kanamycin = 4 |

**Materials and methods**

*2.1. Murine model*

On the first day post-infection, the uninfected and infected control groups received phosphate-buffered saline (PBS), while the ellagic acid high-dose and low-dose groups were administered ellagic acid aqueous solutions at doses of 20 mg/kg and 10 mg/kg, respectively. The AMP treatment group received an AMP aqueous solution at a dose of 10 mg/kg. On days 0, 1, 3, 5, and 7 of the treatment periods, fresh fecal samples were collected from each group of mice. The samples were weighed, mixed with PBS, and thoroughly homogenized using a low-temperature grinder. Following appropriate dilution, the samples were inoculated onto enterococcal agar plates supplemented with 8 µg/mL VAN to monitor VRE growth in the gastrointestinal tract. One day after the administration of the seventh dose, the mice were euthanized. The cecum and ileum tissues were excised, weighed, mixed with PBS, and thoroughly homogenized using a cryogenic grinder. The tissue homogenates were then diluted and transferred to enterococcal agar plates for incubation at 37 ℃ for 48 hours. The total number of colonies in each sample was subsequently counted.

*2.2*. *Analysis of Gut Microbiota*

The purity and concentration of the extracted DNA were evaluated by agarose gel electrophoresis. Subsequently, the DNA was diluted to a concentration of 1 ng/μL in a centrifuge tube. PCR amplification was conducted using the diluted genomic DNA as a template, in conjunction with barcode-containing specific primers, Phusion® High-Fidelity PCR Master Mix with GC Buffer (New England Biolabs), and a high-efficiency, high-fidelity enzyme to ensure optimal amplification efficiency and accuracy. Library preparation was conducted using the TruSeq® DNA PCR-Free Sample Preparation Kit, and the library was quantified using both Qubit fluorometry and quantitative PCR (qPCR). Following successful qualification, sequencing was performed on the NovaSeq6000 platform. Bioinformatics tools such as Trimmomatic were employed to remove low-quality sequences, primers, and adapter sequences, thus enhancing data quality. Subsequently, DADA2 software was utilized for denoising and splicingthe sequences to generate accurate ASVs or operational taxonomic units (OTUs) for downstream analysis. Species annotation of OTU/ASV sequences was conducted using the Mothur method and the SILVA SSUrRNA database, with a similarity threshold ranging from 0.8 to 1.0. This process yielded taxonomic information at various levels, including phylum, class, order, family, genus, and species. The counts at each taxonomic level were subsequently tabulated. Species richness and evenness were calculated using the phyloseq and vegan packages in R. LDA combined with LEfSe analysis was conducted using the LEfSe software. Functional prediction based on the Kyoto Encyclopedia of Genes and Genomes database was performed using Phylogenetic Investigation of Communities by Reconstruction of Unobserved States 2.

*2.3.* *Analysis of SCFAs constituents*

The samples were analyzed using GC with an Agilent DB-FFAP capillary column (30 m × 250 μm × 0.25 μm). The temperature program for the GC was set as follows: the initial temperature was set to 90 ℃, then increased at a rate of 10 ℃/min to 160 ℃, followed by a further increase at 40 ℃/min to 240 ℃, which was held for 5 minutes. Helium was used as the carrier gas at a flow rate of 1.0 mL/min. A quality control (QC) sample was inserted into the sample queue after every specified number of experimental samples to monitor and evaluate the stability and reproducibility of the system. MS was conducted using an Agilent 5977B MSD mass spectrometer under the following conditions: inlet temperature 250 ℃, ion source temperature 230 ℃, transmission line temperature 250 ℃, and quadrupole temperature 150 ℃. Electron impact ionization (EI) was employed with an electron energy of 70 eV, and the analytes were detected in selected ion monitoring (SIM) mode. The supernatant was carefully collected and transferred to a sample vial for gas chromatography-mass spectrometry (GC-MS) analysis. Standard solutions were prepared by serial dilution of the stock standard solution to generate a series of calibration standards. Each calibration standard was processed following the same sample preparation protocol and analyzed using the GC-MS system, with the same method as above.

*2.4.* *Mechanism verification*

Weigh 50 mg of cecal tissue stored at -80 ℃ and transferred into a 1.5 mL nuclease-free centrifuge tube containing 1 mL of pre-chilled Buffer RL1. Place the tube in an ice bath and homogenize the tissue thoroughly using an electric grinder until a uniform suspension is achieved. Add 20 µL of Proteinase K, mixed thoroughly, and incubate the sample at 56 ℃ for 15 minutes. Following incubation, centrifuge the mixture at 12,000 rpm for 5 minutes at 4 ℃. Total RNA was subsequently extracted using the Servicebio® kit according to the manufacturer's instructions. The RNA concentration was measured using an ultramicro spectrophotometer. RNA samples were quantified by reverse transcription and real-Time PCR following the protocol of the PrimeScript™ FAST RT reagent Kit with gDNA Eraser. The Ct values of each PCR reaction were obtained using LightCycler® 96 software. The ΔCt value was calculated by subtracting the Ct value of the internal reference gene from that of the target gene. The ΔΔCt value was determined by subtracting the mean ΔCt of the control group from the ΔCt of the experimental group. Finally, the relative expression changes of the target genes in the experimental group compared to the control group were calculated using the formula 2^-ΔΔCt^.

*2.5. Mechanism verification and Biochemical indices of the mouse cecum*

**Table S3 Primer information for the target gene**

| **Target product** |  | **Oligonucleotide sequence (5’-3’)** |
| --- | --- | --- |
| *GPR41* | F | CTTCTTTCTTGGCAATTACTGGC |
|  | R | CCGAAATGGTCAGGTTTAGCAA |
| *GPR43* | F | CTTGATCCTCACGGCCTACAT |
|  | R | CCAGGGTCAGATTAAGCAGGAG |
| *IL-1β* | F | GCAACTGTTCCTGAACTCAACT |
|  | R | ATCTTTTGGGGTCCGTCAACT |
| *IL-6* | F | TAGTCCTTCCTACCCCAATTTCC |
|  | R | TTGGTCCTTAGCCACTCCTTC |
| *TNF-α* | F | CCCTCACACTCAGATCATCCT |
|  | R | GCTACGACGTGGGCTACAG |
| *NF⁃κB p65* | F | ATGGCAGACGATGATCCCTAC |
|  | R | TGTTGACAGTGGTATTTCTGGTG |
| *β-actin* | F | GAGACCTTCAACACCCCAGC |
|  | R | ATGTCACGCACGATTTCCC |
